# Targeting mutant p53 stabilization for cancer therapy

**DOI:** 10.3389/fphar.2023.1215995

**Published:** 2023-07-12

**Authors:** Jiajian Wang, Wenjun Liu, Lanqing Zhang, Jihong Zhang

**Affiliations:** ^1^ Medical School, Kunming University of Science and Technology, Kunming, China; ^2^ Yunnan Province Clinical Research Center for Hematologic Disease, Kunming, China

**Keywords:** cancer, mutant p53, stabilization, target, degradation

## Abstract

Over 50% cancer bears TP53 mutation, the highly stabilized mutant p53 protein drives the tumorigenesis and progression. Mutation of p53 not only cause loss-of-function and dominant-negative effects (DNE), but also results in the abnormal stability by the regulation of the ubiquitin-proteasome system and molecular chaperones that promote tumorigenesis through gain-of-function effects. The accumulation of mutant p53 is mainly regulated by molecular chaperones, including Hsp40, Hsp70, Hsp90 and other biomolecules such as TRIM21, BAG2 and Stat3. In addition, mutant p53 forms prion-like aggregates or complexes with other protein molecules and result in the accumulation of mutant p53 in tumor cells. Depleting mutant p53 has become one of the strategies to target mutant p53. This review will focus on the mechanism of mutant p53 stabilization and discuss how the strategies to manipulate these interconnected processes for cancer therapy.

## 1 Introduction

The tumor suppressor p53 plays a central role in tumor prevention and therapeutic response. A variety of biological process is regulated by p53 such as cell cycle arrest, senescence and apoptosis (Molchadsky et al., 2010; Bieging et al., 2014). However, p53 is mutated in more than 50% of tumor tissues, and most p53 mutations are missense mutations that occur within the DNA binding structural domain (DBD) of p53 (Olivier et al., 2010; Hainaut and Pfeifer, 2016; Vaddavalli and Schumacher, 2022). The p53 mutations not only lose their original tumor suppressor function, but also profoundly remodel the tumor cell transcriptome in a gain-of-function (GOF) effect which participates in tumorigenesis, proliferation, migration and drug resistance (Muller and Vousden, 2014; Aschauer and Muller, 2016; Stein et al., 2019). The aberrant accumulation of mutant p53 in tumors is an important molecular basis for GOF (Jethwa et al., 2018). On the one hand, mutated p53 is disabled from its negative feedback regulatory loop with E3 ubiquitin ligase and preventing the degradation through the ubiquitin-proteasome pathway in a timely and effective manner, thus accumulating abnormally in tumor cells (Haupt et al., 1997; Midgley and Lane, 1997). Meanwhile, other biomolecules such as a member of the anti-apoptotic factor Bcl-2 family (BGA2/BAG5), or the heat shock protein family (Hsp40, Hsp70 and Hsp90), are involved to regulate mutant p53 stability, for example, E3 ubiquitin ligase MDM2 and Hsp70 induce conformational changes in mutant p53 cells, and Hsp90 is recruited by BGA2 to assemble in the mutant p53 status (Wiech et al., 2012; Yue et al., 2016). In addition, the mutant p53 on its own structural stability hinders degradation, and p53 mutation exacerbates its own temperature-sensitive protein de-folding, and the adhesion sequences buried in the hydrophobic core of p53 are exposed to the protein surface (Ishimaru et al., 2003; Walerych et al., 2012; Soragni et al., 2016). In this process, mutant p53 forms a complex with wild-type p53 and its homologous family members p63 and p73, which hinders degradation while promoting tumor development with dominant-negative effects (DNE) and GOF effects (Xu et al., 2011; Aubrey et al., 2018). Stabilization of mutant p53 is an important driver for abnormal metabolic reprogramming in tumor cells, therefore, understanding the molecular mechanism of mutant p53 stabilization and depleting the mutant p53 in cancer is an important therapeutic strategy.

## 2 Abnormal accumulation and gain of oncogenic function of mutant p53

**FIGURE 1:**
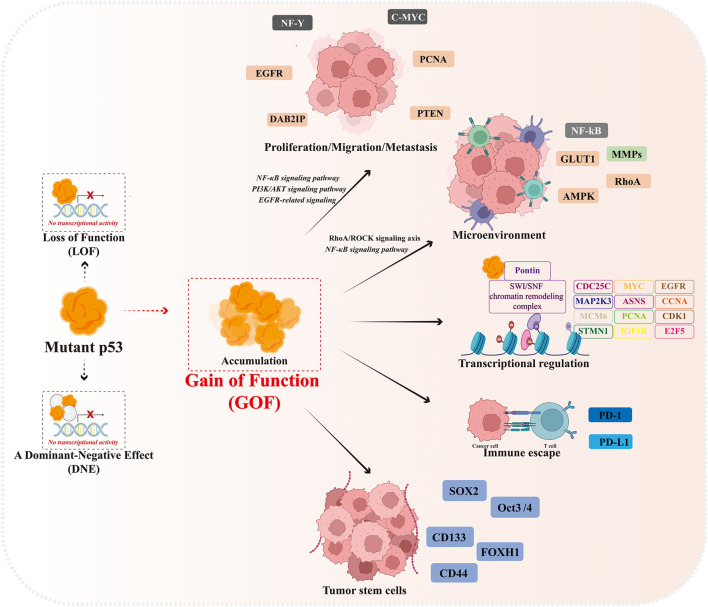
Gain-of-function effect of mutant p53.

Mutant p53 protein undergoes extensive constitutive stabilization in tumors and is closely related to the malignancy, poor prognosis and recurrence of cancer (Brosh and Rotter, 2009; Suh et al., 2011; Alexandrova et al., 2015). The accumulation of mutant p53 in tumors contributes to its functional oncogenic properties. A study provides strong evidence that elimination of stable mutant p53 in the absence of wild-type p53 induces strong cytotoxicity and suppresses tumorigenesis *in vivo* and *in vitro* while boosting survival time in mice by 37% (Alexandrova et al., 2015). Abnormal accumulation of p53 protein is the main feature that distinguishes most tumor cells carrying mutant p53 from normal cells. In addition to losing its own tumor suppressor function (LOF), highly accumulated mutant p53 in tumor cells is able to form a complex with wild-type p53 and inhibit the function of wild-type p53 and MDM2-mediated ubiquitination in a dominant-negative effect (DNE) manner. In addition, mutant p53 gains oncogenic activity in a gain-of-function (GOF) effect, promoting malignant tumor progression, invasion, metastasis and chemoresistance (Figure 1) (Zhou et al., 2019; Dolma and Muller, 2022).

Mutant p53 has a longer half-life than wild-type p53, allowing it to accumulate at high levels in the nucleus, which lead to genomic and transcriptomic alterations in tumor cells. Mutant p53 is incapable to bind to wild-type p53 DNA response element (RE) and disabled the downstream gene expressions and functions, however, p53 mutants trans-activates the expression of other target genes to regulate their GOF properties, such as MYC, CXCL1, PCNA, MAP2K3, CCNA, CCNB, CDK1, CDC25C, ASNS, E2F5, MCM6, IGF1R, STMN1, and EGFR and tumor cell proliferation and migration (Freed-Pastor and Prives, 2012).

At the same time, mutant p53 can also reshape the life activities within tumor cells by binding to other interacting proteins, involving cell proliferation and migration, metabolic reprogramming, tumor microenvironment and immune escape, and cell stemness maintenance (Cordani et al., 2016b). Mutant p53 promotes epidermal growth factor receptor (EGFR) and integrin cell surface translocation by interaction with rab-coupled proteins, activating EGFR-related signaling and promoting cell growth, while mutant p53 activates PI3K/AKT signaling pathway and PD-L1 overexpression through transcriptional inhibition of PHLPP2 expression and binding to DAB2IP, promoting tumor growth and immune escape (Muller et al., 2009; Valentino et al., 2017; Vokes et al., 2022). The mutant p53 activates the RhoA/ROCK signaling axis in tumor cells to promote translocation of GLUT1 (glucose transporter protein 1) to the plasma membrane, thereby enhancing the Warburg effect (Zhang et al., 2013). In addition, mutant p53 activates the NF-κB signaling pathway to induce the expression of intracellular pro-inflammatory factors and the recruitment of chemokines, thereby maintaining the inflammatory state of the tumor microenvironment and regulating tumor-stromal cell crosstalk to promote tumor invasion and migration (Alvarado-Ortiz et al., 2020; Garancher et al., 2020). It has been further reported that mutated p53 abrogates the immunopreventive effect of PD-1 by upregulating IL17 signaling and depleting CD8 cells in the tumor microenvironment, resulting in immune escape of tumor cells (Wang et al., 2021). Meanwhile, the expression of stemness factors such as Klf4, Oct4, Sox2, and c-Myc induced by mutant p53 contributes to the maintenance of the phenotype of tumor stem cells (CSCs). In addition, mutant p53 promotes suppression of chromatin state by increasing the expression level of H3K27me3, which triggers self-renewal of hematopoietic stem cells (Sarig et al., 2010; Chen et al., 2019; Murakami et al., 2019). In tumor samples expressing mutant p53, high expression of CD44 and ALDH correlated with tumor invasion, further emphasizing the role of mutant p53 in promoting the expansion and self-renewal of subpopulations of CSCs(Solomon et al., 2018).

## 3 Mutant p53 stabilization and strategies to destabilize

**FIGURE 2:**
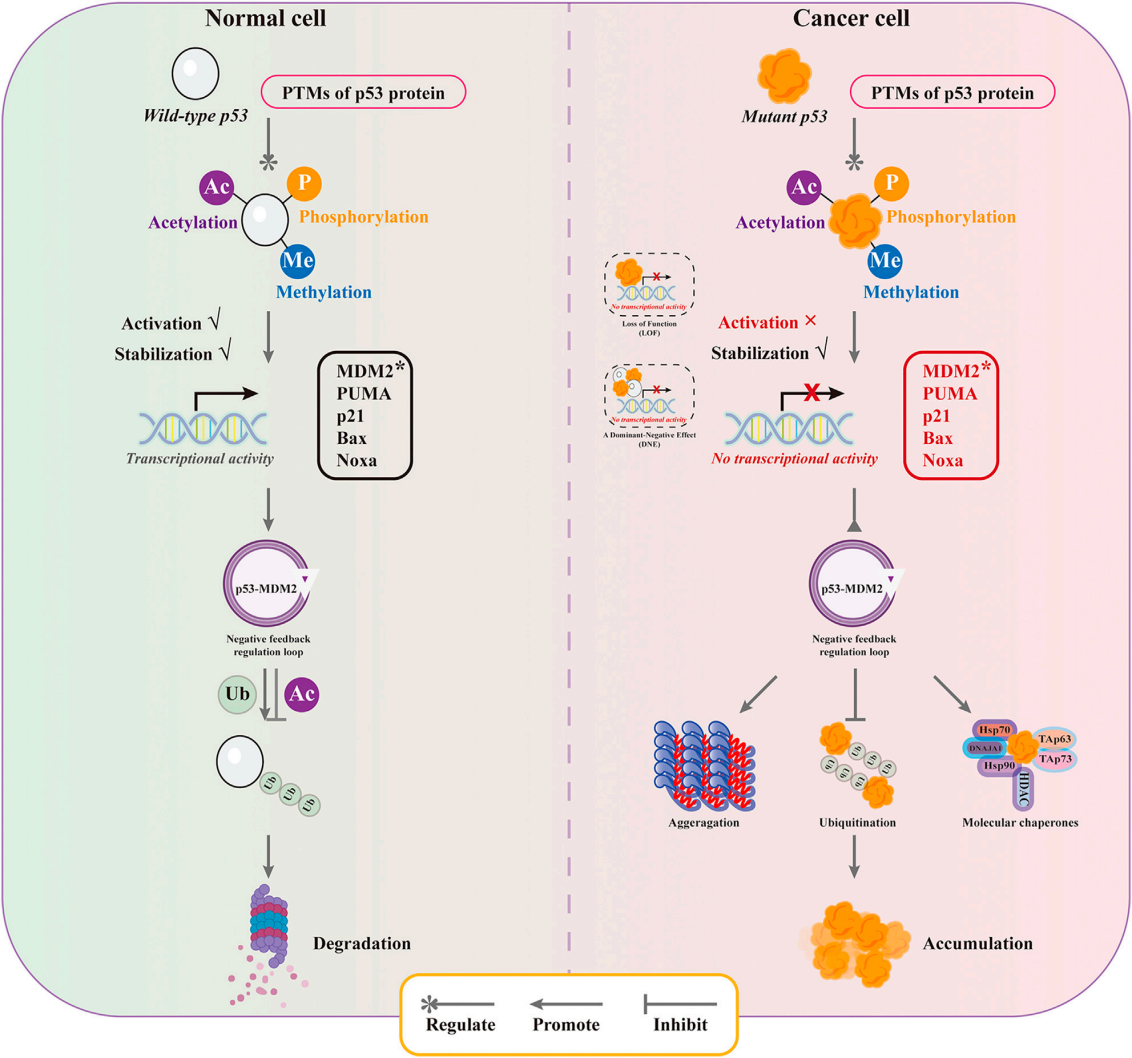
Mechanism of stabilization of mutant p53.

**FIGURE 3:**
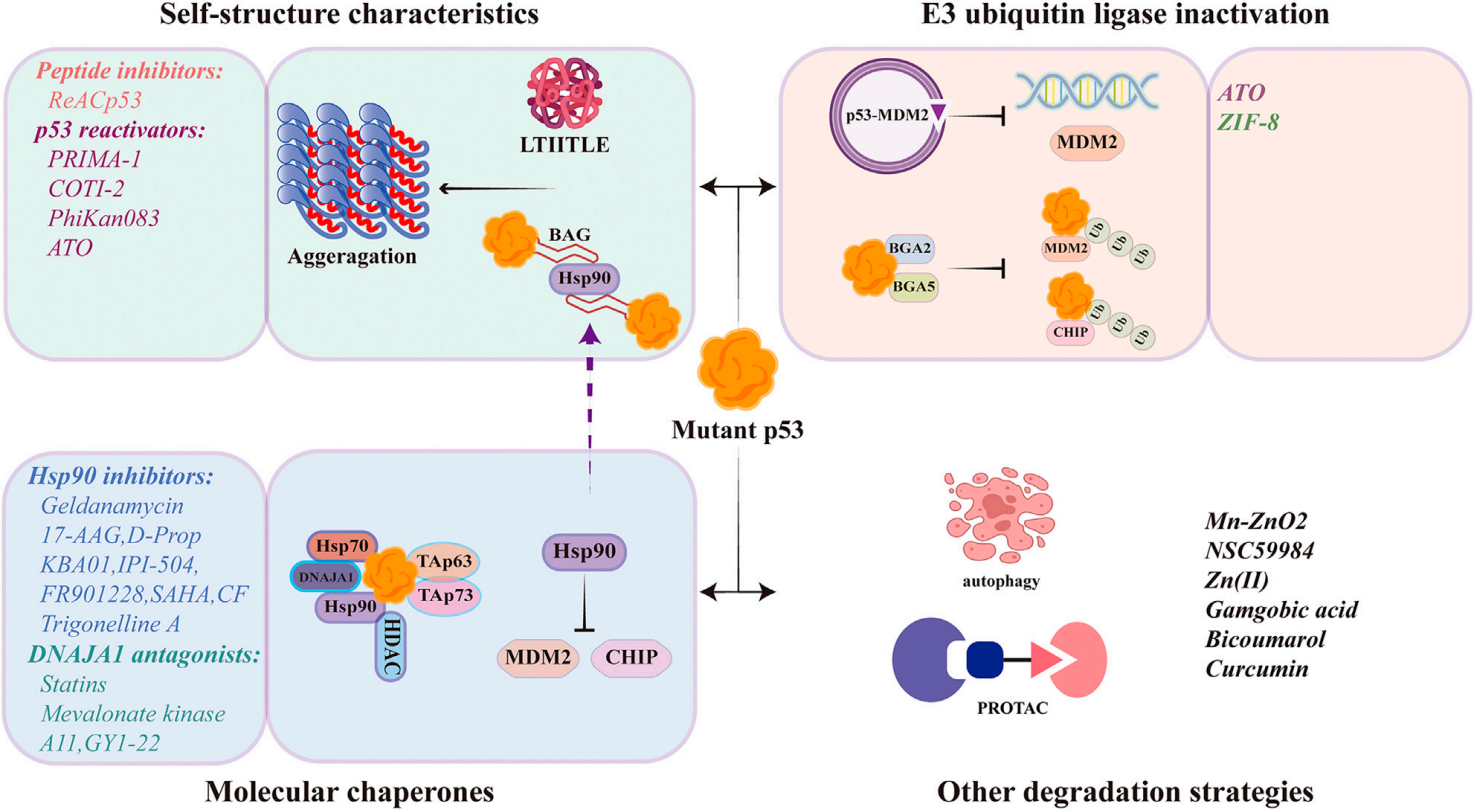
Therapeutic strategy for targeted degradation of mutant p53.

The abnormal accumulation of mutant p53 is closely related to the drug resistance and poor prognosis. Understanding the mechanisms of mutant p53 stabilization and targeted strategies are of great positive significance for tumor treatment and prevention. The main mechanisms of mutant p53 stabilization include three aspects: the properties of mutant p53’s own protein structure, the regulation of E3 ubiquitin ligase, and the mediation of molecular chaperones (Figure 2 and Figure 3).

### 3.1 The properties of mutant p53’s own protein structure

The properties of the p53 protein’s own structure contribute to the formation and stabilization of p53 higher-order polymers. Under physiological conditions, wild-type p53 binds to DNA as a homotetramer in the presence of a tetrameric structural domain, during which the state of p53 shifts from folding-defolding-refolding. Since the majority of mutant p53 is a missense mutation that occurs in its DNA-binding structural domain. The most mutant p53 retains the same tetrameric structural domain as wild-type p53, mutant p53 is able to form heterotetramers with wild-type p53. In addition to inhibiting the function of wild-type p53 in a dominant-negative manner, the formation of heterotetramers also forms stable complexes present in cells in a highly interactive state (folding and defolding) with p53 mutantion. Compared to wild-type p53, mutant p53 has a lower solubilization temperature and stronger interaction between the folded and unfolded states, resulting in mutant p53 being highly susceptible to protein misfolding and accumulation in cells at 37°C. The specific property of the temperature sensitive p53 protein confers an abnormal stability of mutant p53, which is fundamentally tends to aggregate. In previous knowledge, it has been assumed that the stability is not caused by intrinsic features of mutant p53, but rather driven by external tumor-specific events (Schulz-Heddergott and Moll, 2018). However, mutant p53 aggregates give a new definition of mutant p53 stabilization by its intrinsic features. Folded p53 proteins have more flexible conformational shifts and de-folding properties after mutation, inducing mutant p53 aggregation and stabilization in tumor cells. In addition to its own nucleation assembly, the easily aggregated mutant p53 mediates the bioisolation of various tumor suppressors (including wild-type p53, p63, and p73) in the cells, forming a relatively large complex (Xu et al., 2011). This complex is unable to enter the gated channels of the proteasome, preventing to be degraded and therefore stable within the cells (Rodriguez et al., 2012).

The increased propensity for mutant p53 aggregation is mainly achieved by exposing the aggregation nucleation sequence of the hydrophobic core of the p53 DNA binding structural domain. Using the ZipperDB algorithm, Goldschmidt et al. deduced that the DBD of p53 contains several adhesion fragments known as stereospecific zippers, which are also known as high fibrillation propensity fragments, with residues 252–258 identified as the most aggregation-prone fragments, called LTIITLE structures (Soragni et al., 2016). Subsequently, Xu et al. (2011) demonstrated the importance of LTIITLE for p53 aggregation and that the introduction of the aggregation inhibitory mutation I254 effectively suppressed the degree of aggregation of p53 structural mutants in cells (Wang and Fersht, 2017). It is undeniable that the property of temperature-sensitive protein confers flexibility and versatility in the physiological function of p53 protein structure, and the structural instability caused by missense mutations greatly enhances this property of p53. The p53 core structural domain melts at temperatures slightly above body temperature, allowing it to achieve a delicate balance between correct and incorrect folding. However, this balance is very easily disrupted by mutations that promote the formation of aggregates (de Oliveira and Silva, 2017). The Fersht team used differential scanning calorimetry (DSC) and spectroscopic analysis to show that most mutants of the p53 core structural domain are denatured by at least 50% at physiological temperatures (Wang and Fersht, 2015).

Compared with wild-type p53, the increased solvent accessible surface area (SASA) (Li et al., 2020), increased loop flexibility (Langenberg et al., 2020; Li et al., 2020) and exposed backbone hydrogen bonds (BHBs) (Fernández and Scheraga, 2003; Cino et al., 2016) of mutant p53 increase the frequency of protein unfolding and folding transitions, raising the possibility of protein misfolding. Notably, p53 interacts with Hsp70 and MDM2 to produce a “pseudo-aggregation” structure with β-amyloid characteristics, thus maintaining the stability of the p53 mutant (Wiech et al., 2012). Similarly, structurally unstable p53 mutants exert GOF properties by inducing co-aggregation of wild-type p53 and its homologous family members p63 and p73, and inhibition of aggregation therefore restores the activity of p53 wild-type function and homologous family members by eliminating GOF (Li and Prives, 2007; Costa et al., 2016).

Mutation of p53 exacerbates its interaction between protein folding and de-folding, resulting in protein misfolding. Misfolded mutant p53 is highly susceptible to nucleation and assembly, forming amyloid aggregates. Therefore, inhibition of mutant p53 aggregation induces an active conformational shift of mutant p53 and promotes the degradation of mutant p53. Currently, various designer peptides or small molecules are used to destabilize mutant p53. Designer peptides aim to inhibit mutant p53 aggregation, direct changes in p53 protein homeostasis, and promote protein refolding to degradation induction. Small molecules reactivate mutant p53 and restore its wild-type function to some extent also exhibiting a reduction in p53 expression levels.

#### 3.1.1 Peptide inhibitors

ReACp53 is a cell membrane-penetrating peptide, that is, the most prominent therapeutic approach for targeting mutant p53 aggregation, which is designed based on the p53 adhesion sequence (LTIITLE structure). The researchers designed the LTRITLE sequence (ReACp53) with an arginine substitution at the position based on the side chain arrangement in the LTIITLE structure (Soragni et al., 2016). ReACp53 can inhibit aggregation by covering the adhesion sequence of mutant p53 exposed to the protein surface through the structural complementary property. ReACp53 functions in the aggregation phase of p53 dynamic equilibrium to rescue mutant p53 function, and ReACp53 possesses good targeting to mutant p53 and exhibits good anti-tumor activity *in vivo* and *in vitro*. In primary prostate cancer (PCa), ReACp53 inhibits mutant p53 aggregation and degrades mutant p53 in a manner that increases protein ubiquitination, while increasing the sensitivity of tumor cells to chemotherapeutic agents (Zhang et al., 2020; Neal et al., 2021).

#### 3.1.2 p53 reactivators

Maintaining the mutant p53 native state is an important way to inhibit aggregation (Pedrote et al., 2018). In the last two decades of research, several mutant p53 reactivators have been developed for the targeted treatment of mutant p53. The compounds such as PRIMA-1, PK083, and COTI-2 have entered preclinical studies. PRIMA-1 (2,2-dihydroxymethyl-1-azabicyclo [2.2.2]octan-3-one) is a mild alkylating agent, which can be converted to active Michael receptor methylene quinuclidinone (MQ) *in vivo* to covalently bind to cysteine residues in the p53 core region, mainly Cys124 and Cys277, thereby promoting the refolding of p53 protein into an active conformation and exert anti-tumor function (Zhang et al., 2018). PRIMA-1-mediated refolding of mutant p53 causes the adhesion sequences exposed on the protein surface to be greatly reburied in the hydrophobic core of p53 protein, thus reducing the nucleation propensity of mutant p53 and inhibiting the formation of mutant p53 aggregates. While effectively inhibiting the formation of mutant p53 aggregates, PRIMA-1 further restores the ubiquitination of mutant p53 and promotes the degradation of mutant p53. Further data suggest that the cytotoxicity caused by PRIMA-1/APR-246 is related to the degradation of p53 after p53 refolding (Russo et al., 2010; Jaskova et al., 2020). (Table 1).

**TABLE 1:** Clinical trials targeting degradation of P53 in cancer therapy, sourced from the ClinicalTrials.gov database (https://clinicaltrials.gov/ct2/home).

Role	Compound	Disease	Phase	NCT number	Mechanism of actin
Self-structure characteristics	PRIMA-1	Oesophageal carcinoma	Phase Ib/II	NCT02999893	Reactivation of mutant p53 and inhibition of aggregation
		High-grade serous ovarian cancer	Phase Ib/II	NCT02098343	
		AML or MDS	Phase II	NCT03931291	
		MDS	Phase III	NCT03745716	
	COTI-2	Advanced or recurrent malignancies	Phase I	NCT02433626	Reactivation of mutant p53
	ATO	Refractory cancer	Phase II	NCT04695223	Metamorphic agents, Promotion of mutant p53 ubiquitination
		Refractory solid tumors	Phase II	NCT04869475	
Molecular chaperones	Geldanamycin	Epithelial ovarian cancer, fallopian tube cancer, primary peritoneal cancer	Phase I/II	NCT02012192	Hsp90 inhibitor
	IPI-504	Non-Small Cell Lung Cancer	Phase II	NCT01362400	Hsp90 inhibitor
		Prostate Cancer	Phase II	NCT00564928	
	Atorvastatin	Colorectal carcinoma	Phase II	NCT04767984	Disruption of DNAJA1 interaction with mutant p53
	Lovastatin	Ovarian Cancer	Phase II	NCT00585052	Disruption of DNAJA1 interaction with mutant p53
	SAHA	Advanced cancers	Phase I	NCT02042989	HDAC6 inhibitor
Other degradation strategies	Zn(II)	Metastatic colorectal cancer	Phase II	NCT03898102	Autophagy agonists

### 3.2 E3 ubiquitin ligase inactivation with mutant p53 stabilization

The ubiquitination modifications are one of the major influences on the accumulation of mutant p53 in tumor cells, which is determined by both the altered ubiquitination efficiency and the dysregulation of the p53-MDM2 negative feedback regulatory loop. In normal cells, wild-type p53 undergoes a series of post-translational modifications such as phosphorylation, acetylation, methylation and glycosylation to regulate p53 activity under the stimulation of stress conditions. The acetylation of Lys residues in p53 protein to stabilize p53 and promote its accumulation in the nucleus and enhance p53 transcription, and methylation modifications to enhance p53 stability and promote its occurrence and accumulation. Post-translational modifications inhibit MDM2-mediated p53 ubiquitination and enhance p53 stability and activity, allowing p53 to be highly expressed and exert tumor suppressive functions (Kruse and Gu, 2009; Levine and Oren, 2009). Persistent high expression of p53 is detrimental to cell survival. A highly ordered intracellular regulatory mechanism facilitates the resolution of one of the problems, namely, ubiquitination of p53 promotes deacetylation of p53. Since MDM2 is a classical p53 downstream target gene and its expression is regulated by p53, high expression of MDM2 occurs after p53-activated transcription. High expression of MDM2 leads to p53 deacetylation and destabilization and promotes p53 degradation via the ubiquitin-proteasome pathway. The negative feedback regulatory loop formed between p53 and MDM2 ensures the dynamic equilibrium of p53 under stress conditions, maintaining it in a low expression state (Hock and Vousden, 2010). However, compared with wild-type p53, the ubiquitination modification of mutant p53 is far from sufficient to effectively induce its degradation. On the one hand, the distortion of the conformation of the DBD region of mutant p53 alters the binding mode of p53 to MDM2 with temperature-sensitive factors. The mutant p53 DBD provides a secondary binding site for MDM2 and facilitates the interaction between MDM2 and mutant p53 with the involvement of the MDM2 nomenclature domain, resulting in a much lower ubiquitination efficiency of mutant p53 by MDM2 than wild-type p53 (Lukashchuk and Vousden, 2007). On the other hand, it is associated with the malfunction of the p53-MDM2 negative feedback regulatory loop. In the p53-MDM2 negative feedback regulatory loop, mutant p53 cannot effectively activate MDM2 transcription due to loss-of-function effect (Muller et al., 2008). Moreover, p53 proteins with structural mutations have high affinity to the central acidic structural domain of MDM2 and can interfere with RING-mediated ubiquitination to inhibit the E3 ubiquitin ligase activity of MDM2 (Yang et al., 2019). Although mutant p53 is observed to be regulated by post-translational modifications similar to wild-type p53 in tumor cells, the fact that mutant p53 interferes with the self-activation of MDM2 molecules contributes to reduced mutant p53 ubiquitination, resulting in increased stabilization and decreased destabilization of mutant p53 in tumor cells, ultimately leading to aberrant accumulation of mutant p53 in tumor cells and promoting tumor development (Ahn et al., 2014).

The chaperone-associated E3 ubiquitin ligase CHIP (the C-terminus of Hsc70-interacting protein) is a key component of the molecular chaperone complex and usually marks abnormal and misfolded polypeptides for degradation (Ahmed et al., 2012). Consistently, CHIP induces proteasomal degradation of wild-type p53 and mutant p53 associated with Hsc70 and Hsp90 chaperones (Esser et al., 2005). However, this was extended in a study in which refolded mutant p53 was ubiquitinated and degraded through a different pathway, distinguishing from MDM2, and CHIP-mediated ubiquitination was specific to mutant p53 (Lukashchuk and Vousden, 2007). Moreover, the abnormal mutant p53 ubiquitination was related to other factors. It was found that the BAG structural domains contained in the anti-apoptotic factors Bcl-2 family members BAG2 and BAG5 are able to interact with mutant p53, and this BAG-mutant p53 interaction blocks the binding of MDM2 and CHIP to mutant p53, which in turn promotes the accumulation and GOF of mutant p53 in tumorigenesis, as is indicated that co-expression of BAG5 in H1299 cells in a dose-dependent manner significantly reduced the interactions of mutant p53-MDM2 and mutant p53-CHIP (Arakawa et al., 2010; Yue et al., 2015; Yue et al., 2016).

In addition, other ubiquitin ligases play important roles in regulating mutant p53 stability, such as TRIM21, Pirh2, bTrCP1, WWP1, and RNF128. The E3 ubiquitin ligase TRIM21 (Tripartite motif containing-21) as a critical E3 ubiquitin ligase of mutant p53 was found to bind specifically to p53 R175H (Liu et al., 2023), resulting in ubiquitination and degradation of mutant p53 to suppress mutant p53 GOF in tumorigenesis. TRIM21 shows low expression in a variety of tumors and is closely associated with poor prognosis of tumor patients (Zhou et al., 2018; Zhou et al., 2021). However, the low expression of TRIM21 was found to be significantly correlated with the high expression of mutant p53 in the correlation analysis (Liu et al., 2023). This result suggests that although TRIM21 degrades mutant p53 in a R175H mutant-specific binding manner, its low expression level in tumor tissues carrying mutant p53 leads to the accumulation of p53 R175H mutants and promotes the GOF effect of mutant p53. Pirh2 (RING-H2 protein), WWP1(WW domain-containing ubiquitin E3 ligase 1), RNF128(RING finger protein 128) all have E3 ubiquitin ligase protein members that regulate wild-type p53 homeostasis through ubiquitination modifications, but they also exhibit E3 ubiquitin ligase activity against mutant p53 and are involved in regulating mutant p53 stabilization (Laine and Ronai, 2007; Chen et al., 2013; Yan et al., 2014).

Since E3 ubiquitin ligase regulates the homeostasis of mutant p53 by the ubiquitin-proteasome pathway, therefore, alterations of the E3 ubiquitin ligase can be the strategy to manipulate mutant p53 levels.

#### 3.2.1 Arsenic trioxide (ATO)

ATO induces the expression of the E3 ubiquitin ligase Pirh2, which promotes the physical interaction of Pirh2 with mutant p53 protein to form polyubiquitination and induces mutant p53 degradation. Ectopic expression of Pirh2 or ATO treatment significantly reduced the level of mutant p53. Ectopic expression of Pirh2 in combination with ATO treatment further reduced the level of mutant p53. The ability of Pirh2 E3 ligase to degrade mutant p53 was confirmed to be enhanced by ATO treatment (Yan et al., 2014). In addition, ATO restoration of mutant p53 elucidates the mechanism of mutant p53 degradation from a novel perspective, arsenic in ATO binds to a cryptocysteine triplet to rescue structural p53 mutants, and arsenic binding stabilizes the DNA-binding loop-sheet-helix motif and the entire β-sandwich fold, conferring thermal stability and transcriptional activity to the p53 mutant. Promoting mutant p53 degradation from a refolding perspective (Chen et al., 2021). (Table 1).

#### 3.2.2 Novel nanomaterials

Zeolite imidazolate framework-8 (ZIF-8), a non-toxic and biocompatible nanomaterial consisting of zinc ions as metal nodes and 2-methylimidazolate as a junction molecule, exhibited extensive mutant p53 degradation. This performance was attributed to the material characteristics of ZIF-8, where ZIF-8 catabolism in acidic endosomes sustained elevated intracellular Zn and decreased intracellular GSH:GSSG ratio which in turn led to enhanced mutant p53 glutathionization and ultimately polyubiquitination and mutant p53 degradation (Zhang et al., 2021); Cerium oxide nanoparticles induce a K48 ubiquitination-dependent degradation of broad-spectrum mutant p53 protein, that is, dependent on dissociation of mutant p53 protein from heat shock protein Hsp90/70 and an increase in reactive oxygen species (ROS) (Zhang et al., 2023).

### 3.3 Molecular chaperones and mutant p53 stabilization

Molecular chaperones are involved in important intracellular life events chaperone proteins of the Hsp70 and Hsp90 families are key players in cellular events and protect aberrant proteins from degradation. The key premise for mutant p53 to have the GOF effect is to stabilize protein through Hsp90/Hsp70/Hsp40 chaperone mechanism, thus protecting mutant p53 from being degraded by ubiquitination of MDM2 and other E3 ligands (Muller et al., 2008; Wiech et al., 2012). The activity of Hsp70 and Hsp90 is regulated by the co-chaperone Hip protein, which enhances the interaction of Hsp70 with the guest protein by stabilizing the ADP-bound form (Dahiya et al., 2019).

Hsp40 isoform DNAJA1 was identified as a structurally mutated p53 binding protein that promotes stabilization of misfolded conformational mutant p53 proteins through physical interactions and protects mutant p53 from CHIP-mediated degradation. DNAJA1 contains three other conserved regions, including a glycine/phenylalanine-rich structural domain, a zinc finger structural domain, and the C-terminus. The C-terminus of the DNAJA1 CAAX motif is critical for its ability to stabilize mutant p53, which regulates the farnesylation and inhibition of DNAJA1 farnesylation promotes mutant p53 degradation and inhibits mutant p53-driven oncogenesis. DNAJA1 controls the fate of misfolded mutant p53 via the mevalonate pathway (Parrales et al., 2016), which blocks E3 ubiquitin ligase CHIP-mediated degradation of mutant p53 in a competitive binding manner and enhances mutant p53 stability (Xu et al., 2019). Molecular docking data showed that Glu198 and Ala138 of mutant p53 and Pro84 and Lys125 of DNAJA1 were essential for the binding. Therefore, DNAJA1 can be used as an important drug target for targeted degradation of mutant p53 (Tong et al., 2021).

Hsp90 plays a key role in the conformational stabilization and maturation of mutant oncogenic signaling proteins. These include steroid hormone receptors, receptor tyrosine kinases (HER-2), signaling kinases (Bcr-Abl, Akt, and Raf-1) and mutant p53 (Neckers and Workman, 2012). Many mutant p53 proteins are impaired in their conformationally sensitive core structural domains and form abundant stable complexes with Hsp90 in tumor cells. This stable mutant p53-specific interaction with Hsp90 chaperones in cancer cells is hypothesized to be related to the aberrant stabilization of mutant p53. On the one hand, Hsp90 binding in the mutant p53-MDM2-Hsp90 ternary complex hides the Arf binding site on MDM2 and blocks the function of MDM2; On the other hand, the presence of the Hsp90 complex also inhibits CHIP activity (Li et al., 2011b). Histone deacetylases (HDAC) are enzymes that regulate the deacetylation of many histone and non-histone proteins, thereby affecting a wide range of cellular processes. Histone deacetylase 6 (HDAC6) is a unique histone deacetylase with two functional catalytic structural domains (DD1 and DD2) and a ZnF-UBP structural domain (ubiquitin binding structural domain, BUZ) that regulates many biological processes including gene expression, cell motility, immune response and degradation of misfolded proteins (Liu et al., 2021). Hsp90 is one of lysine deacetylase substrates of HDAC6, the Hsp90/HDAC6 chaperone mechanism protects mutant p53 from degradation by CHIP and MDM2. In conclusion, the stable interaction of mutant p53 high expression and activated Hsp90 effectively inhibits MDM2 and CHIP activity, leading to their abnormal stabilization. Disrupting the Hsp90 system by depleting the Hsp90 core protein or pharmacologically inhibiting Hsp90 ATPase activity with a competitive ATP pocket inhibitor releases mutant p53 from the complex and reactivates endogenous MDM2 and CHIP for mutant p53 degradation. However, interestingly, a recent study has uncovered a new mechanism by which Hsp90 stabilizes mutant p53, again providing new ideas for targeting molecular chaperones to degrade mutant p53. Members of the BGA family of proteins are able to induce mutant p53 stabilization by binding preferentially to p53 structural mutants in tumor cells carrying mutant p53. Further Hsp90 is involved in the formation of higher-order polymers of the BAG2-mutant p53 complex under the recruitment of BAG2, facilitating the propagation and maintenance of the aggregates. Silencing of BAG2 and inhibition of Hsp90 activity effectively suppressed proliferation and metastasis under mutant p53 aggregation reinforcing the role of Hsp90 in regulating mutant p53 stability (Huang et al., 2023).

#### 3.3.1 Hsp90 inhibitors

Pharmacological reduction of mutant p53 stability has extensively investigated. In particular, inhibition of the heat shock protein 90 (Hsp90) and histone deacetylase 6 (HDAC6) chaperone complexes to stabilize mutant p53 remains the most studied approach to mutant p53 destabilization. The Hsp90 inhibitor geldanamycin reduced mutant p53 expression in cancer cell lines and simultaneously refolded mutant p53 into a more wild-type like conformation. The same 17-AAG (17-allylamino-17-demethoxygeldanamycin) inhibited the ATPase activity of Hsp90 disrupting the complex of mutant p53 and Hsp90, and released mutant p53 and reactivated endogenous MDM2 and CHIP to degrade mutant p53 (Roh et al., 2013). IPI-504 (a novel Hsp90 inhibitor) inhibited the recruitment of BAG2 to Hsp90 and reduced \mutant p53 aggregates (Huang et al., 2023). Notably, the mutant p53 protein, which is stabilized and mediates cellular oncogenic addiction through interaction with the chaperone Hsp90, becomes unstable after D-Prop treatment. The phosphorylation of Hsp90 by PKA and its interaction with mutant p53 is reduced by D-Prop, releasing mutant p53 for proteasomal degradation (Barra et al., 2021). KBA01 is a natural compound of alkaloid origin from boxwood that releases mutant p53 from the protein complex by inhibiting Hsp90 activity while destabilizing the HSF1-mutant p53-Hsp90 complex. This process results in enhanced interaction of mutant p53 with MDM2 and CHIP, promoting ubiquitination and proteasomal degradation of mutant p53 (Wang et al., 2020).

Stable complex formation between Hsp90 and its mutant p53 client inhibits E3 ligases MDM2 and CHIP. SAHA shows preferential cytotoxicity in mutant p53 cancer cells by destabilizing mutant p53 through inhibition of the HDAC6-Hsp90 chaperone axis, this releases mutant p53 and enables its MDM2 and CHIP mediated degradation (Li et al., 2011a). HDAC6-selective inhibitor, A452, increased wild-type p53 levels by destabilizing MDM2, but decreased mutant p53 by inducing MDM2 and inhibiting Hsp90-mutant p53 complex formation. Interestingly, HDAC6 levels inversely correlated with p53 acetylation at lysines 381/382 associated with p53 functional activation (Ryu et al., 2017). Resveratrol, an HDAC6 inhibitor, increases the acetylation level of Hsp90 and decreases Hsp90 activity, and disrupts mutant p53 binding to Hsp90, leading to mutant p53 destabilization. In fact, inhibitors such as FR901228 (Shetty et al., 2021), Trigonelline A (Tanveer et al., 2023), and Colletofragarone A2 (CF) (Sadahiro et al., 2022) all cause Hsp90-dependent mutant p53 depletion.

#### 3.3.2 DNAJA1 antagonists

The mevalonate pathway contributes to mutant p53 stabilization. Specific reduction of mevalonate 5-phosphate by statins or mevalonate kinase knockdown induces CHIP ubiquitin ligase-mediated nuclear export, ubiquitination, and mutant p53 degradation by impairing the interaction of mutant p53 with the DNAJA1. Knockdown of DNAJA1 also induced CHIP-mediated mutant p53 degradation, and its overexpression antagonized statin-induced mutant p53 degradation (Parrales et al., 2016).

Some compounds provide unique solutions for the interaction of DNAJA1 with mutant p53, which bind to DNAJA1 via tyrosine 7 (Y7), lysine 44 (K44) and glutamine 47 (Q47), and creat a competitive relationship with mutant p53, allowing mutant p53 to free itself from the stabilizing mechanism of DNAJA1, enhancing the E3 ubiquitin ligase for ubiquitination of mutant p53 for degradation (Nishikawa et al., 2022).

GY122 screening from a drug library specifically disrupted the binding between mutant p53 and DNAJA1. GY1-22 significantly reduced p53 R172H protein levels and activated gene p21 expression, GY1-22 not only eliminated mutant p53 but also restored the tumor suppressive function of wild-type p53 by eliminating the dominant negative effect of mutant p53, further combination of atorvastatin significantly reduced mutant p53 protein levels (Tong et al., 2021). (Table 1).

### 3.4 Regulation of redox homeostasis and autophagic degradation of mutant p53

In normal cells, p53 plays an important role in ROS detoxification, maintaining low levels of oxidants; whereas tumor cells exhibit elevated levels of ROS (Cordani et al., 2020). Mutant p53 proteins can sustain ROS production, thereby promoting chemoresistance and proliferation in cancer cells. Cells expressing mutant p53 show reduced expression of ALDH4A1 and decreased NRF2 activity, leading to defects in ROS detoxification and cell survival (Dolgacheva et al., 2019). Mutant p53 can further stabilize its own expression by regulating redox homeostasis to obtain pro-cancer functions such as drug resistance. Further, mutant p53 isoforms have been shown to regulate various antioxidant cellular systems or enzymes in different ways compared to wild-type p53 functions. The NOX4 protein is the catalytic subunit of the NADPH oxidase complex that catalyzes the reduction of molecular oxygen to various ROS (Neckers and Workman, 2012), mutant p53 enhances NOX4 expression with a modest increase in ROS to promote cancer cell proliferation and survival. Where NADPH stabilizes mutant p53 in a physically binding manner (Asher et al., 2002).

Inhibition of AMPK phosphorylation leads to stimulation of the mTOR pathway. Expression of mutant p53 inhibits BECN1, DRAM1, ATG12, SESN1/2, suppresses AMPK phosphorylation and reduces autophagy. Mutant p53 proteins have been shown to promote defective autophagy in cancer cells, which in turn may lead to the accumulation of aberrant mitochondria, resulting in ROS induction, genomic instability, and cancer development and progression (Agarwal et al., 2016; Cordani et al., 2016a; Cordani et al., 2018). The inhibition of autophagy by mutant p53 is likewise responsible for its prolonged half-life and abnormal accumulation.

Many small molecules which regulate redox levels in tumor cells and autophagy induce mutant p53 degradation. Mn-ZnO_2_ nanoparticles deliver zinc-manganese double ions and ROS into tumors to regulate the expression and function of p53 protein for precise treatment of p53 mutant tumors. The Mn-ZnO_2_ nanoparticles are able to break down in the weak acidic environment of the tumor and release Zn^2+^ and H_2_O_2_ by Fenton-like reaction, which induce ubiquitination and proteasomal degradation of mutant p53, while releasing Mn^2+^ and increasing ROS levels, thereby activating the ATM-p53-Bax pathway and increasing wild-type p53 levels, thereby inhibiting tumor cell survival and proliferation (Wang et al., 2023).

NSC59984 induced ROS induction and its associated signaling promoted degradation of mutant p53. NSC59984 induced restoration of p53 pathway signaling and degraded mutant p53 protein in colorectal cancer cells expressing mutant p53). NSC59984 induced sustained phosphorylation of ERK1/2 in cancer cells in the ROS-Ras-MEK-ERK2 axis and subsequent degradation of mutant p53 protein via ERK2, at higher ROS levels, phosphorylation of MDM2 induced by NSC59984 bound to mutant p53 (Zhang et al., 2022).

A novel Zn (II) compound induces mutant p53 (R175H) protein degradation via autophagy, Zn(II) restores the ability of wild-type p53 to induce the expression of the p53 target gene DRAM, a key regulator of autophagy (Garufi et al., 2013). Similarly, Gamgobic acid (GA) stimulates mutant p53 degradation and increases the sensitivity of cancer cells to chemotherapeutic agents. GA may induce p53-R280K (MDA-MB-231) and p53-S241F (DLD1) protein degradation via the autophagic pathway (Foggetti et al., 2017). Bicoumarol and curcumin are able to induce p53 degradation by inhibition of NAD(P)H:quinone oxidoreductase 1 (NQO1) activity (Asher et al., 2002; Tsvetkov et al., 2005). (Table 1).

### 3.5 PROTAC

Targeted protein degradation (TPD) is a new therapeutic modality through direct depletion of target proteins, which mainly utilizes two proteostasis mechanisms, the intracellular ubiquitin-proteasome system (USP) and the lysosomal system, to achieve target protein degradation (Xue et al., 2023). PROteolysis Targeting Chimera (PROTAC) is currently the dominant degradation strategy. PROTAC consists of two binding units and a linker unit, one of which recognizes the target protein and the other recruits the ubiquitin ligase (E3 ligase), thereby ubiquitinating and degradation. However, PROTACs targeting p53 mutants have been rarely reported because of the difficulty in finding suitable binders for p53 mutations due to the relatively smooth surface of p53 protein. Recently, a nucleic acid aptamer-based PROTAC has emerged to overcome this dilemma. The PROTAC is composed of a short single-stranded oligonucleotide that binds specifically to the p53 structural mutant R175H as the head to recognize the target protein and a tail that recruits the ubiquitin ligase with thalidomide-O-amido-propynyl group, and is named dp53m-RA. dp53m-RA degrades p53-R175H in a ubiquitin proteasome-dependent manner, but not wild-type p53 or other p53 mutants. Importantly, dp53m-RA inhibited the proliferation and migration of cancer cells specifically harboring the p53-R175H mutation (Chen et al., 2015; Kong et al., 2023).

## 4 Summary and outlook

The p53 missense mutations are present in more than 50% of human tumors and 75% of human tumors with altered transitions, acting as pro-oncogenic factors. The p53 mutations and their aberrant stabilization and accumulation in tumor cells are key factors in the malignant transformation of tumors. The GOF effect caused by mutant p53 stabilization induces tumor-specific dependence and resistance to chemotherapy. Degradation of mutant p53 is effective in attenuating the oncogenic effects induced by the GOF effect of mutant p53 in malignant tumors, especially in cancers carrying heterozygous mutant/wild-type p53 alleles. Therefore, targeted degradation of mutant p53 protein has great potential for improving prognosis, prolonging survival of cancer patients and cancer therapy. The high frequency of p53 mutations in tumors and its intrinsic tumor suppressor function make it a potential promising target for cancer therapy. However, the mutant p53 protein bears relatively smooth surface whithout a ideal drug binding pockets, which stalled the drug development in p53. Currently, strategies for targeted degradation of mutant p53 focus on enhancing mutant p53 ubiquitination, inhibiting aggregate formation and disrupting mutant p53 with other protein complexes. With the technology development, targeted degradation of mutant p53 may be a better strategy to remove their oncogenic effects, such as PROteolysis Targeting Chimera (PROTAC), and Lysosome-targeting chimaeras (LYTAC) and Autophagy-targeting chimera (AUTAC).
