# Supplementary material for: Targeting mutant p53 stabilization for cancer therapy
Source: Front Pharmacol. 2023 Jul 12;14:1215995. doi: 10.3389/fphar.2023.1215995 (PMC10369794; doi:10.3389/fphar.2023.1215995)
Supplement: Supplementary file 1 [file Table1.DOCX]

| Table 1.Clinical trials targeting degradation of P53 in cancer therapy，, sourced from the ClinicalTrials.gov database(https://clinicaltrials.gov/ct2/home) | | | | | |
| --- | --- | --- | --- | --- | --- |
| Role | Compound | Disease | Phase | NCT number | Mechanism of actin |
| Self-structure characteristics | PRIMA-1 | Oesophageal carcinoma | Phase Ib/II | NCT02999893 | Reactivation of mutant p53 and inhibition of aggregation |
|  |  | High-grade serous ovarian cancer | Phase Ib/II | NCT02098343 |  |
|  |  | AML or MDS | Phase II | NCT03931291 |  |
|  |  | MDS | Phase III | NCT03745716 |  |
|  | COTI-2 | Advanced or recurrent malignancies | Phase I | NCT02433626 | Reactivation of mutant p53 |
|  | ATO | Refractory cancer | Phase II | NCT04695223 | Metamorphic agents，Promotion of mutant p53 ubiquitination |
|  |  | Refractory solid tumors | Phase II | NCT04869475 |  |
| Molecular chaperones | Geldanamycin | Epithelial ovarian cancer, fallopian tube cancer, primary  peritoneal cancer | Phase I/ II | NCT02012192 | Hsp90 inhibitor |
|  | IPI-504 | Non-Small Cell Lung Cancer | Phase II | NCT01362400 | Hsp90 inhibitor |
|  |  | Prostate Cancer | Phase II | NCT00564928 |  |
|  | Atorvastatin | Colorectal carcinoma, | Phase II | NCT04767984 | Disruption of DNAJA1 interaction with mutant p53 |
|  | Lovastatin | Ovarian Cancer | Phase II | NCT00585052 | Disruption of DNAJA1 interaction with mutant p53 |
|  | SAHA | Advanced cancers | Phase I | NCT02042989 | HDAC6 inhibitor |
| Other degradation strategies | Zn(II) | Metastatic colorectal cancer | Phase II | NCT03898102 | Autophagy agonists |
